# Individually Rate Corrected QTc Intervals in Children and Adolescents

**DOI:** 10.3389/fphys.2019.00994

**Published:** 2019-08-02

**Authors:** Irena Andršová, Katerina Hnatkova, Kateřina Helánová, Martina Šišáková, Tomáš Novotný, Petr Kala, Marek Malik

**Affiliations:** ^1^Department of Internal Medicine and Cardiology, University Hospital Brno, Faculty of Medicine, Masaryk University, Brno, Czechia; ^2^National Heart and Lung Institute, Imperial College London, London, United Kingdom

**Keywords:** age, sex differences, individual QT/RR patterns, QT/RR hysteresis, QTc interval, QT/RR slope

## Abstract

Accurate evaluation of the appearance of QTc sex differences during childhood and adolescence is intricate. Inter-subject differences of individual QT/RR patterns make generic heart rate corrections inaccurate because of fast resting heart rates in children. The study investigated 527 healthy children and adolescents aged 4–19 years (268 females, 50.9%). All underwent continuous ECG 12-lead monitoring while performing postural changes during a 70-min investigative protocol to obtain QT interval measurements at different heart rates. On average, more than 1200 ECG measurements (QT interval and its 5-min history of preceding RR intervals) were made in each subject. Curvilinear QT/RR regression involving intra-individual correction for QT/RR hysteresis were calculated in each subject. The projection of the QT/RR regressions to the heart rate of 60 beats per minute defined individually corrected QTc intervals. In males, gradual QTc shortening by about 15 ms appeared during the ages of 13–19 years synchronously with the incidence of secondary sex signs (*p* = 0.016). On the contrary, whilst gradual QTc prolongation by about 10 ms appeared in females, it occurred only during ages 16–19 years and was not related to the incidence of secondary sex signs (*p* = 0.18). The study also showed that in children and adolescents, linear QT/RR models fit the intra-subject data significantly more closely than the log-linear models (*p* < 0.001). The study speculates that hormonal shifts during puberty might be directly responsible for the QTc shortening in males but that QTc prolongation in females is likely more complex since it was noted to follow the appearance of secondary sex signs only after a considerable delay.

## Introduction

As recently reviewed in detail (Linde et al., 2018), there are substantial sex differences in many electrophysiology processes and characteristics. While many of the sex differences have important clinical implications, their physiologic origin is frequently insufficiently understood. Among others, as repeatedly observed (Linde et al., 2018), adult pre-menopausal females have higher resting heart rate and longer QTc interval compared to males of the same age. Based on large ECG collections, it was previously suggested that these changes occur during puberty which lead to the conclusion that sex hormones trigger these differences (Rautaharju et al., 1992; Kurokawa et al., 2016). Indeed, the role of hormones was supported by other studies, e.g., by the observation that the sex of the recipient rather than that of the donor influences QTc sex differences after heart transplant (Novotný et al., 2014).

Nevertheless, the previous studies of the development of QTc interval during childhood and adolescence suffered from the problem of inaccurate heart rate correction. It is well known that in adults, the relationship between QT duration and the underlying heart rate is subject-specific with substantial differences between different individuals (Batchvarov et al., 2002) and that generic heart rate corrections (e.g., Bazett, Fridericia, or Framingham formulae) may lead to noticeable QTc inaccuracy if applied to QT intervals measured at heart rate considerably remote from 60 beats per minute (bpm) (Malik et al., 2002; Garnett et al., 2012). While it is not known whether similar inter-subject differences exist in children, fast resting heart rates are well recognized in young children (Sarganas et al., 2017). Generic QT heart rate corrections may therefore be highly imprecise in individual children (Hnatkova et al., 2019) while normative data obtained from population-based regressions between QT intervals and simultaneously measured heart rates (Rautaharju et al., 2014) might be influenced by substantial correction errors (Malik et al., 2019).

These methodological shortcomings impact not only on the physiologic understanding of the QTc development during childhood but may also lead to difficult judgment of QTc interval in borderline clinical cases. Having these problems in mind, we have designed a physiologic study of healthy school-age children and adolescents and recorded their continuous 12-lead electrocardiograms (ECG) during provocative maneuvers. This allowed us to study individual QT/RR profiles and to investigate their development and sex differences during childhood and adolescence.

## Materials and Methods

### Investigated Population

Per protocol, the study investigated healthy children of school age with the aim of obtaining, in both sexes, uniform age distribution between the ages of 6–19 years. The recruitment was organized at six primary (including preparatory years) and secondary schools in northern and southern Moravia offering ECG-based health check. While every child or adolescent who agreed to participate was investigated, the data used in the analysis presented here excluded those who were on repolarization affecting drugs^1^ or on hormonal contraceptives, and those with cardiac abnormality. The study protocol was approved by the Ethics Committee of the University Hospital Brno. All participants (if legally allowed to do so) or their parents or legal guardians gave informed written consent according to the Helsinki declaration.

Standard demographic data were collected in all participants; body mass index was calculated according to the formula *W*/*H*^2^ where *W* is the body weight in kilograms and *H* is body height in meters. Presence of secondary sex characteristics corresponding to recognized standards (Harlan et al., 1979, 1980; Mickey and Brooke, 2019) was detected by a combination of visual inspection and a questionnaire submitted by parents/guardians of the investigated subjects.

### Investigative Protocol

Continuous 12-lead ECG (SEER MC version 2, sampled at 1000 Hz) with electrodes in the Mason-Likar position was recorded in each participant during 70-min provocative postural maneuvering that consisted of supine, sitting, standing, supine, standing, sitting, and supine positions (in this order) each of 10-min duration. The sitting and standing positions were maintained without external support and the position changes were accomplished within less than 20 s.

Participants were investigated in the mid-morning hours in groups of up to 20 subjects of similar ages performing the positional changes at the same time. During the investigation, younger children listened to non-exciting age-appropriate stories, others were investigated in quiet noise-free environment.

To make the study practical, the ECG recordings were started before the provocative maneuvering and terminated afterward. Consequently, the duration of the ECG recordings before and after the provocative maneuvering differed in different subjects. During these times, the subjects were engaged in standard school activities excluding physical education. None of the participants smoked before or during the ECG recording.

### Electrocardiographic Measurements

For the purposes of the present investigation, all ECGs were divided into 10-s segments with 5-s overlap between adjacent segments. In each segment, QRS complexes were identified, and in each lead, representative P-QRS-T beatform was constructed by calculating sample-by-sample medians of superimposed beats. An algebraic composite of threshold, tangent, wavelet, and polynomial interpolation algorithms that provided pre-review QT assessment in previous studies (Malik et al., 2008a, 2012b) was used to obtain automatic QT interval measurements. The same algorithm of this composite method was used in all recordings of this study; i.e., there were no measurement differences between different study subjects and/or between different ECGs of the same subject. Consistency of QT interval measurements between neighboring (non-adjacent) segments was used to eliminate noise-influenced and other problematic QT values. In each subject, all non-eliminated QT measurements were subsequently adjusted by pattern-matching algorithms (Malik et al., 2004; Hnatkova et al., 2009) to ensure that QRS onset and T wave offset patterns of similar morphology were measured consistently.

For each QT interval measurement, individual RR intervals within the measured 10-s segment and in the preceding 5 min were identified and the sequence of their durations obtained. The accurate QRS detection needed for this purpose was based on a combination of automatic QRS detectors (Kohler et al., 2002; Pahlm and Sornmo, 1984; Kors et al., 1986) with visual control and manual corrections where appropriate.

### Heart Rate and QT/RR Hysteresis Correction

Using previously published technology (Malik et al., 2008a, 2012b), individual QT/RR patterns were investigated in each study participant including the curvilinear regression of the relationship. Since the QT interval duration depends on the underlying heart rate rather than on the rate of the immediately preceding heartbeat cycle (Franz et al., 1988), QT/RR hysteresis was taken into account. Hence, as previously published (Malik et al., 2012b), the assessment of QT/RR patterns was combined with subject-specific assessment of the QT/RR hysteresis profiles which were modeled using the exponential decay forms (Malik et al., 2008b).

Curvilinear models of the hysteresis-corrected QT/RR patterns were used to estimate the QT interval duration at RR interval of 1 s (i.e., at heart rate of 60 bpm). This was based on averaging individually corrected QT values of all measurements available in the given subject. The result of this averaging is further called the QTcI interval.

To estimate the importance of QT/RR hysteresis correction, QT/RR patterns were also investigated relating the QT interval measurements to the average of 3 RR intervals in the middle of the 10-s ECG segment in which the QT measurement was made as well as to the average of all RR intervals in this segment. The range of heart rates available in each subject (i.e., the “width” of heart-rate data allowing to study the subject-specific QT/RR pattern) was defined as the difference between the maximum and minimum hysteresis corrected heart rates for which the QT measurements were available.

To compare the hysteresis corrected QT/RR patterns between different study subjects, linear and log-linear regression models were further used in the forms *QT*_*i*_ = β_0_ + β*RR*_*i*_ + ε_*i*_ and *log*⁡*QT*_*i*_ = α_0_ + α*log*⁡*RR*_*i*_ + ε_*i*_, respectively. Here *QT*_*i*_ and *RR*_*i*_ are mutually corresponding QT and RR interval measurements (expressed in seconds) in the given subject and in both formulae, ε_*i*_ are zero-centered normally distributed errors. The standard deviations or the ε_*i*_ errors (i.e., the individual-specific regression residuals) were used to compare the accuracy of both regression models. These models were used since, as well known, they lead to the QT correction formulae *QTc* = *QT* + β(1−*RR*) and *QTc* = *QT*/*RR*^α^.

### Statistics and Data Presentation

Numerical data are presented as mean ± standard deviation. Dependency of ECG measurements on age was investigated using linear regression models that were displayed together with the 95% confidence intervals (CI) of the regression lines. When the age dependency appeared non-linear, averages and standard deviations were calculated and compared in separate age bands <8 years, 7–9 years, 8–10 years, etc. up to 16–18 years, and >17 years (the overlaps were used to obtain sufficient case numbers in each band). Intra-subject differences of the QT/RR regression residuals were assessed by the non-parametric Wilcoxon matched-pair test. Differences between females and males, differences between subjects showing and not showing secondary sex signs, and differences between males or females younger and older than a dichotomy age limit were investigated using non-parametric Kolmogorov–Smirnov test. Dichotomy age limits of 10, 11, …, 16 years were used. Statistical calculations were made in the SPSS Statistics 64-bit version 25 package (IBM, Armonk, NY, United States). *P*-values < 0.05 were considered statistically significant.

## Results

### Population

After the call for participation, 555 subjects (295 females) were enrolled and underwent the investigative protocol. Of these, 27 (4.9%) had to be excluded because of potentially interfering drug therapy, cardiac structural congenital abnormalities (including those with a history of cardiac surgery), cardiac conduction abnormality, and (in one case) sex-transversal procedures. Of the remaining 528 subjects, further one (0.2%) was excluded because of technical failure.

The analysis reported here is thus based on 527 subjects. Of these, 268 were females and 259 were males. Secondary sex signs were observed in 182 (67.9%) and 145 (56.0%) of females and males, respectively. Table 1 shows the characteristics of the investigated population in individual age groups. Supplementary Figure 1 shows that, as expected, the body weight and height was similar between sexes of young age. With increasing age, males became taller and heavier compared to females. Body mass index (Supplementary Figure 2) was also increasing with advancing age but no significant difference between sexes was observed.

**TABLE 1:** Investigated population.

**Age [years]**	**Females**					**Males**				
	***N***	**Sign**	**Height [cm]**	**Weight [kg]**	**ECG**	***N***	**Sign**	**Height [cm]**	**Weight [kg]**	**ECG**
≤7	17	0	115.7 ± 5.2	20.5 ± 2.8	868 ± 353	15	0	119.1 ± 6.9	24.7 ± 7.4	1019 ± 348
7–8	14	1	128.1 ± 9.8	26.1 ± 3.9	1113 ± 501	14	0	128.5 ± 6.0	25.9 ± 3.5	1017 ± 389
8–9	23	1	132.5 ± 5.4	28.4 ± 4.1	1012 ± 455	18	0	135.4 ± 5.7	31.9 ± 5.6	1113 ± 301
9–10	15	5	139.2 ± 7.0	33.7 ± 6.3	1459 ± 501	11	0	141.9 ± 7.8	36.1 ± 8.6	1075 ± 442
10–11	23	7	144.2 ± 5.6	36.5 ± 5.6	1548 ± 501	24	1	145.0 ± 7.1	38.0 ± 8.1	1200 ± 489
11–12	15	12	149.9 ± 7.8	38.4 ± 7.5	1365 ± 493	24	9	150.3 ± 6.9	44.6 ± 9.7	1235 ± 431
12–13	22	18	159.1 ± 5.6	48.3 ± 10.3	1181 ± 419	29	20	160.6 ± 10	52.9 ± 12.5	1449 ± 430
13–14	23	22	160.8 ± 8.0	48.2 ± 6.1	1146 ± 401	20	16	163.5 ± 6.6	49.6 ± 9.2	1295 ± 602
14–15	22	22	166.2 ± 5.2	58.3 ± 12.7	1122 ± 435	16	13	173.7 ± 7.3	59.9 ± 10.7	1514 ± 388
15–16	34	34	165.8 ± 5.3	56.7 ± 7.5	1011 ± 448	33	31	177.8 ± 6.9	68.1 ± 9.7	1346 ± 501
16–17	28	28	167.6 ± 5.4	58.3 ± 6.7	1087 ± 340	20	20	180.0 ± 7.7	72.7 ± 8.6	1359 ± 478
17–18	11	11	173.5 ± 4.6	64.3 ± 12.6	1298 ± 672	16	16	182.2 ± 6.4	70.6 ± 9.2	1234 ± 378
>18	21	21	165.9 ± 7.3	57.0 ± 8.3	1054 ± 292	19	19	178.5 ± 6.5	70.4 ± 11.9	1519 ± 511

*For each age bin, the table shows the number (N) of investigated females and males, the number of those showing secondary sex signs (Sign), their heights and weights in centimeters and kilograms, respectively, the number of ECG measurement per subject (ECG). Data are shown as mean ± standard deviation.*

Altogether 19 subjects (3.6% of the investigated populations; 13 females and 6 males) have not completed the investigation protocol because of pre-syncopal episodes, nausea, or vomiting. Nevertheless, in all these subjects, sufficient ECG data were collected before such side-effects occurred. No ECG data potentially influenced by the side-effects were included in the analysis.

The ECG data reported are based on 642,003 measurements of the QT interval and of its 5-min RR interval history. On average, 1218 measurements were made per subject (Table 1).

Supplementary Tables 1, 2 summarize electrocardiographic measurements (as presented in subsequent sections in more detail) in individual age groups.

### QT/RR Hysteresis

**FIGURE 1:**
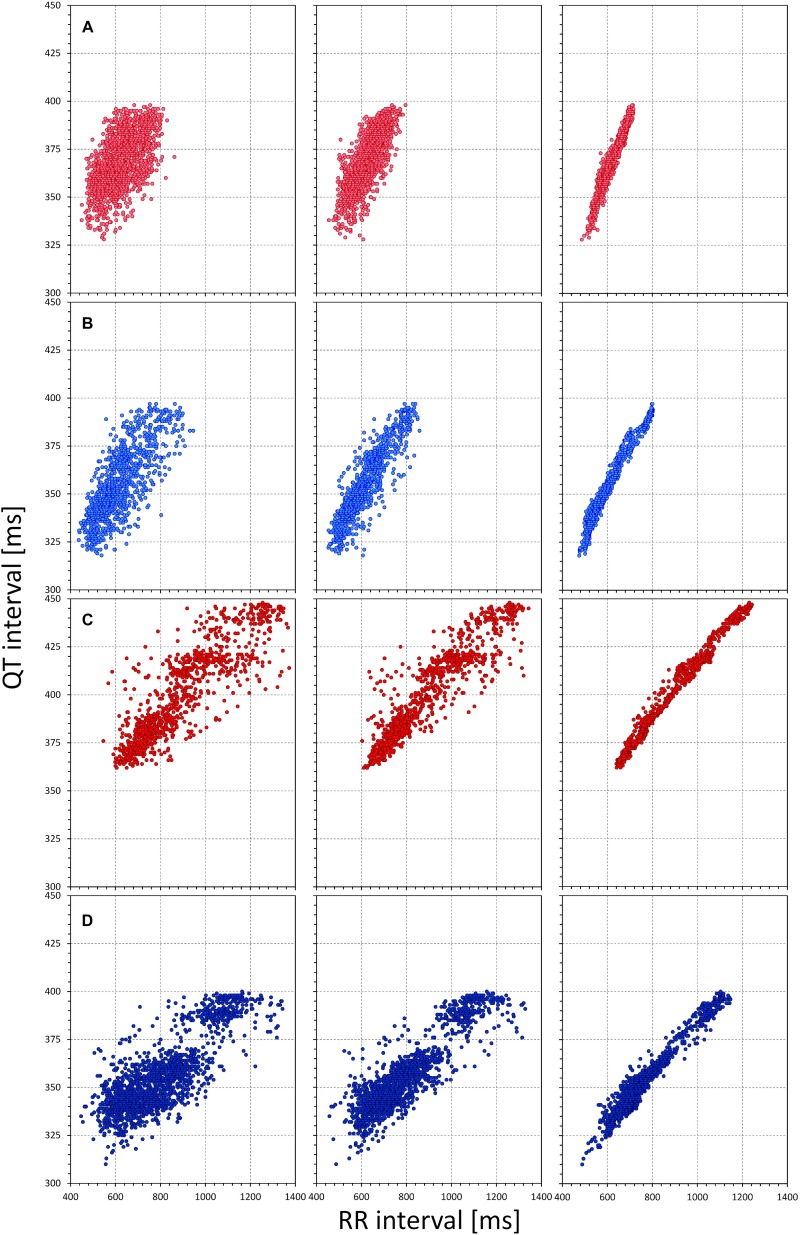
The figure shows examples of comparisons of QT/RR relationship using different RR interval expressions. Each row of panels corresponds to one subject. In each subject, the QT interval measurements are the same but the panels on the left relate the QT intervals to the averages of three RR intervals, the panels in the middle to 10-s RR interval averages, and the panels of the right to the RR interval values obtained from the 5-min histories of the QT interval measurements by individual QT/RR hysteresis profiles. Note the increase of the regression fit from the left panels to the right panels. Cases **(A–D)** correspond to a female aged 7.4 years, a male aged 7.7 years, a female aged 18.7 years, and a male aged 18.7 years, respectively.

**FIGURE 2:**
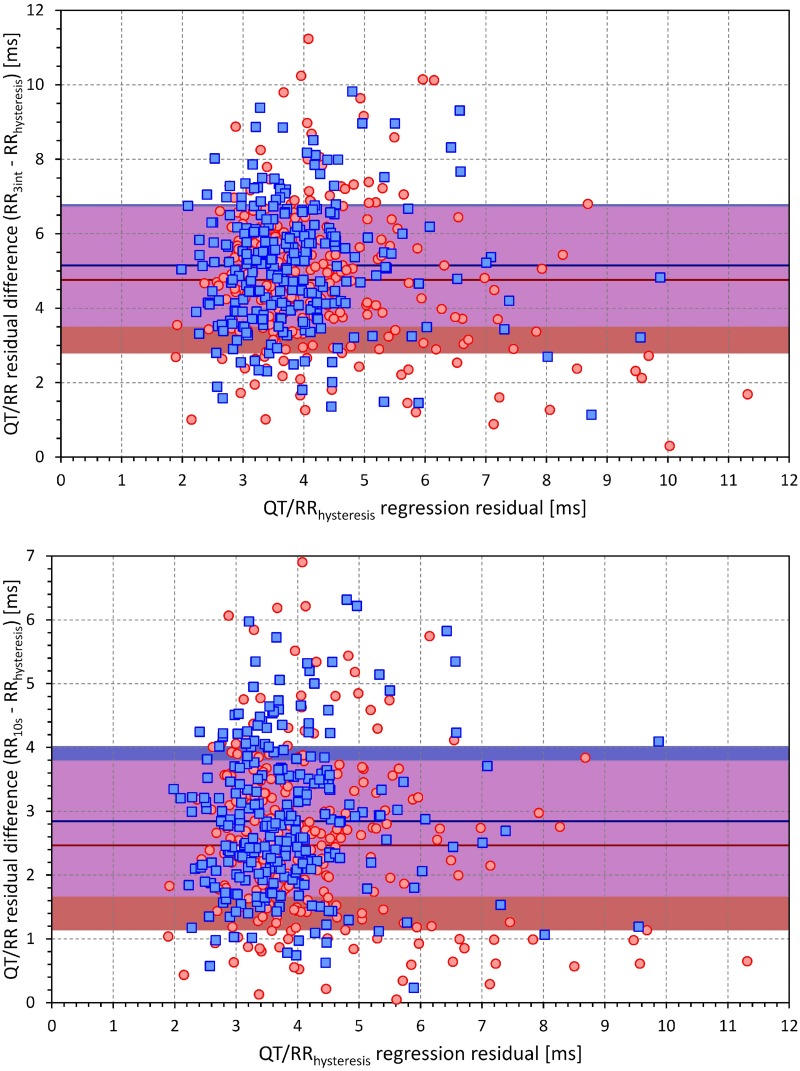
Scatter diagrams between the curvilinear QT/RR regression residuals involving QT/RR hysteresis correction (horizontal axes) and the increases in the residuals when using three RR interval averages **(top)** and 10-s RR interval averages **(bottom)** instead of QT/RR hysteresis correction. Red circles and blue squares correspond to female and male subjects, respectively. Dark red and dark blue lines are means of the residual increase in females and males, respectively. The light red and light blue bands show the intervals of mean ± standard deviation of the sex-specific residual increases. The violet bands are the overlaps between the standard deviation bands of both sexes.

Figure 1 shows examples documenting that the inclusion of QT/RR hysteresis led to substantially more tight relationship between the measured QT intervals and the underlying heart rate. This was verified by the regression residuals. With the incorporation of QT/RR hysteresis, the curvilinear QT/RR regression residuals (i.e., the spread of the QT data around the curvature of the relationship) were 4.59 ± 2.60 ms and 3.87 ± 1.16 ms in females and males, respectively (*p* < 0.001). With the RR intervals averaged from the 10-s ECG segments, these residuals were 7.05 ± 2.40 ms and 6.73 ± 1.64 ms, whilst with the averages of three RR intervals, the residuals increased to 9.34 ± 2.56 ms and 9.04 ± 1.97 ms. All comparisons of these data were highly statistically significant (*p* < 0.001 for all). Figure 2 shows that the increase of the QT/RR regression residuals from the hysteresis corrected relationship to either RR interval averages over 10 s or RR interval averages over three heart cycles occurred in every study participant.

**FIGURE 3:**
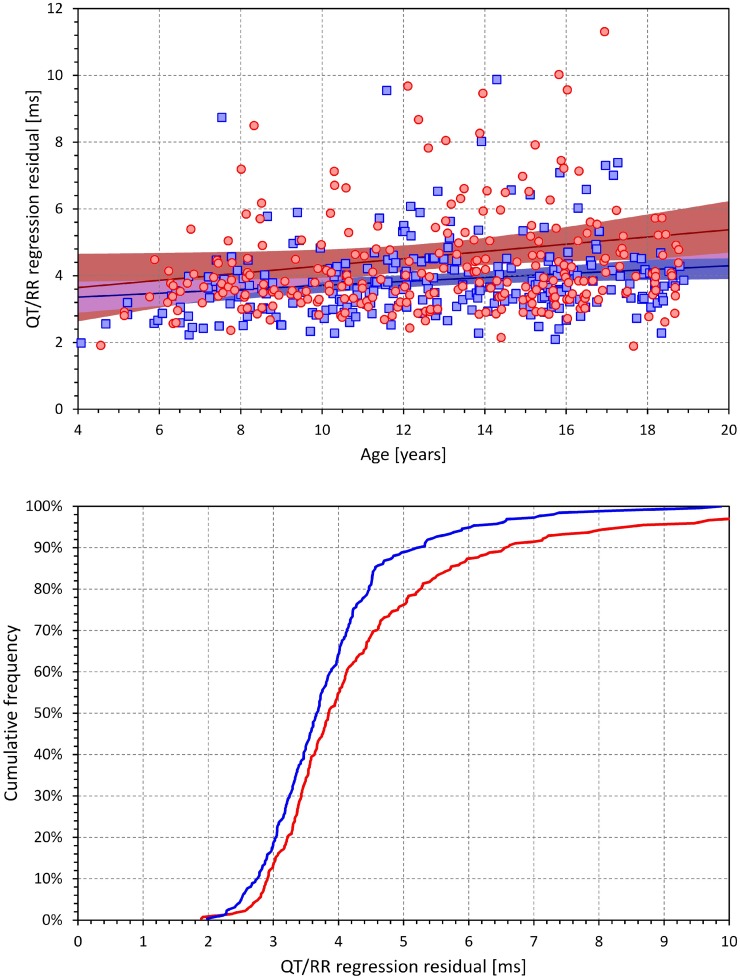
The **(top)** panel show the age dependency of the curvilinear QT/RR regression residuals. The red circles and blue squares correspond to the female and male subjects, respectively. The dark red and dark blue lines are linear regressions in females and males, respectively; the light red and light blue areas are the 95% confidence bands of the sex-specific linear regression lines. The violet areas are the overlaps between the confidence bands of the regression lines of both sexes. The **(bottom)** panel shows the cumulative distributions of QT/RR regression residuals in female (bold red line) and male (bold blue line) subjects.

The top panel of Figure 3 shows that in both females and males, the QT/RR regression residuals (hysteresis corrected) increased with increasing age. In both sexes, the increase was modest with an increase of 0.11 ms per year in females and 0.06 ms in males (both statistically significant, *p* < 0.01). The bottom panel of Figure 3 shows cumulative distributions of the QT/RR regression residuals in both sexes and demonstrates that both in females and males, the curvilinear relationship between the QT interval duration and the underlying heart rate was very tight (note that in both sexes, the median QT/RR residual was below 4 ms).

**FIGURE 4:**
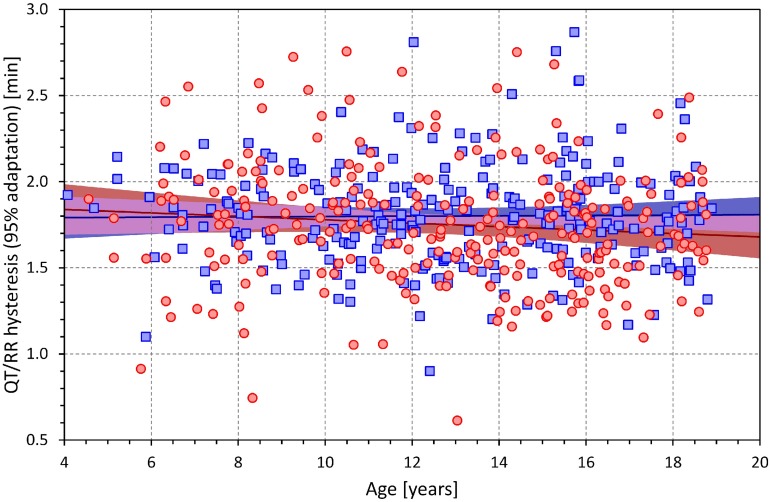
Age dependency of the intra-subject QT/RR hysteresis time constants (i.e., time intervals needed for 95% adaptation of QT interval duration after heart rate change). The layout and symbol definition are the same as in Figure 3.

The extent of the QT/RR hysteresis is standardly expressed by its time constant defined as the time interval required for 95% of the adaptation of QT interval to occur after a heart rate change. The dependence of this hysteresis time constant on the age of study subjects is shown in Figure 4. The extent of the QT/RR hysteresis was independent of age and, on average, very close to the constant of 2 min.

Since in every subject, QT intervals were more closely related to the hysteresis corrected RR interval values compared to the other possible RR interval expressions, the measurements involving QT/RR hysteresis correction were used in the subsequent parts of the study.

### Heart Rate Changes

**FIGURE 5:**
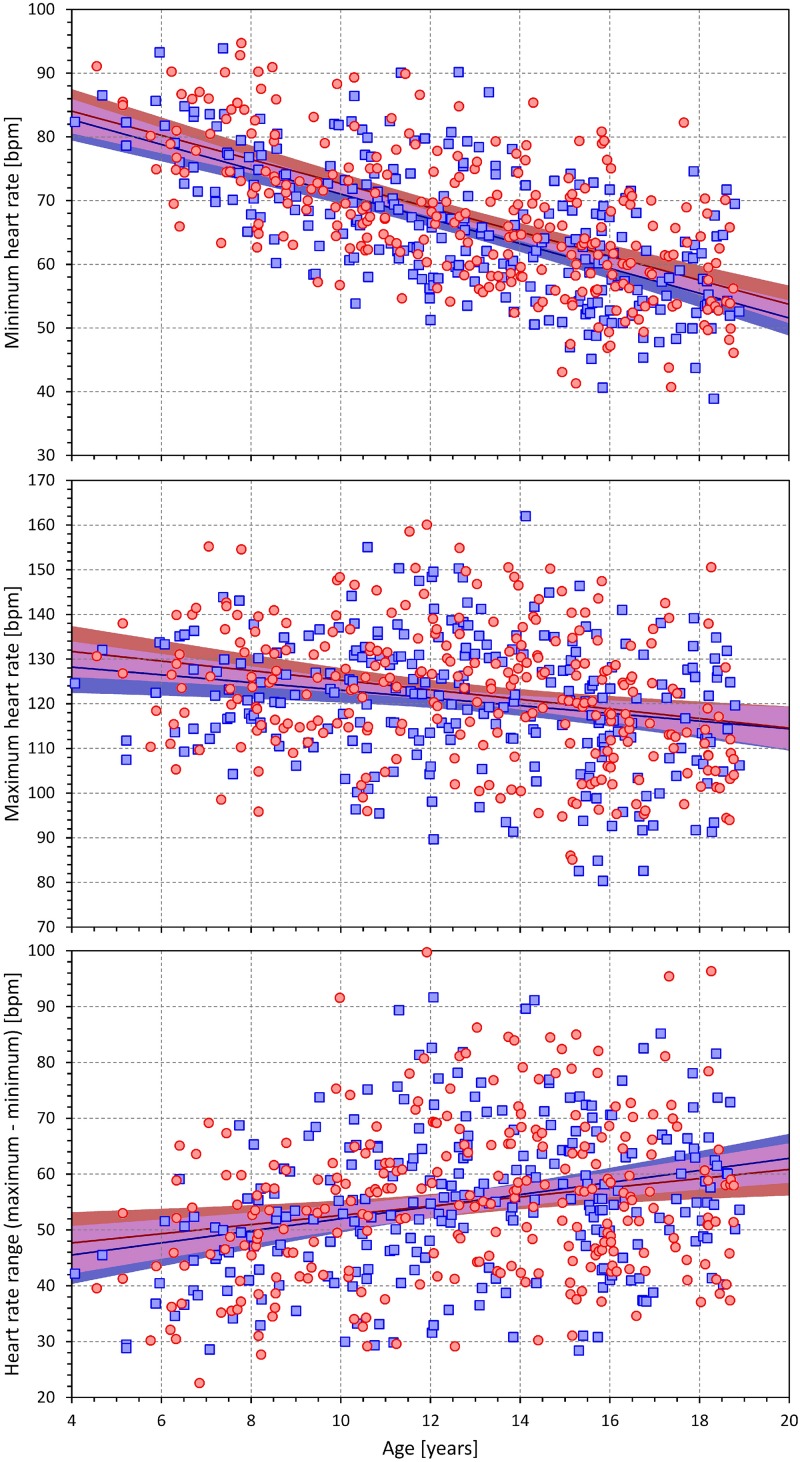
Scatter diagrams between the age and minimum heart rate **(top)** maximum heart rate **(middle)** and the range between the minimum and maximum heart rate **(bottom)**. The layout and symbol definition in each panel are the same as in Figure 3.

Figure 5 shows the age-dependency of slowest and fastest heart rates at which the QT intervals were measured as well as of the intra-subject heart rate ranges over which the individual QT/RR patterns were assessed.

As expected, both the slowest and fastest heart rates were higher in younger children compared to adolescents (in both sexes, the minimum heart rates were decreasing by 1.9 bpm per year of age, *p* < 0.001; the decrease of maximum heart rate was approximately 50% shallower but still highly statistically significant, *p* < 0.001). Nevertheless, the bottom panel of Figure 5 shows that in all study subjects, the heart rate spreads of QT/RR patterns were substantial. The individual QT/RR patterns were thus accurately defined.

### QTcI Interval

**FIGURE 6:**
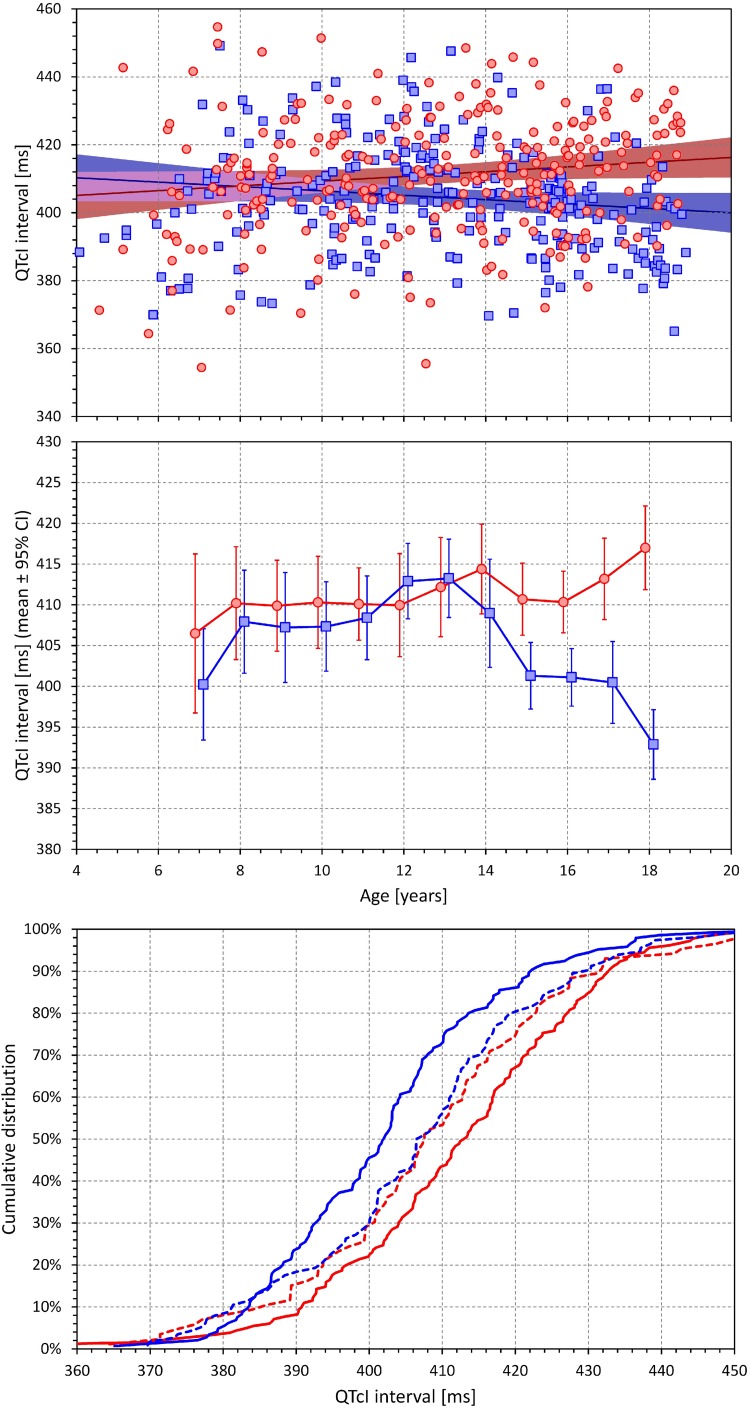
The **(top)** panel shows the scatter diagram between age and QTcI interval (see the legend of Figure 2 for symbol explanations). The **(middle)** panel shows the averages of QTcI intervals in age bands <8 years, 7–9 years, 8–10 years, etc. up to 16–18 years, and >17 years (each shown approximately at the middle age of the band). The error bars are the corresponding standard deviations. The red and blue marks correspond to the females and males, respectively. The **(bottom)** panel shows the cumulative distributions of QTcI intervals in subjects without (dashed lines) and with (full lines) secondary sex signs. The red and blue lines again correspond to females and males, respectively.

Figure 6 shows the relationship of QTcI intervals to age which was one of the principal results of the study. In the linear regression analysis (top panel of Figure 6), there was significant of QTcI prolongation with advancing age in females (0.70 ms per year, *p* = 0.02) and significant QTcI shortening with advancing age in males (0.64 ms per year, *p* = 0.03).

Nevertheless, as shown in the middle panel of Figure 6, the change with age was highly non-linear. No clear differences between the sexes were found up till the age of approximately 12–13 years. From that age on, QTcI interval was gradually decreasing in males. In females, no obvious QTcI change was seen up till the age of approximately 16 years following which, the QTcI interval increased substantially. In the highest age bin of >17 years, the average QTcI differences between the sexes was 24.1 ms.

The surprising late onset of the QTcI change in females was confirmed by the comparison of QTcI distributions in subjects showing and not showing secondary sex signs, as demonstrated in the bottom panel of Figure 6. There was no difference between the distributions in females and males without secondary sex signs. The comparison between those with and without secondary sex signs led to only a non-significant trend in females (*p* = 0.181) whilst in males, the same comparison resulted in a clear statistical significance (*p* = 0.016). The sex difference between females and males showing the secondary sex signs was highly significant (*p* < 0.001).

When dichotomizing the population (females and males separately) into those younger and older than 10, 11, 12, …, 16 years, the QTcI difference between older and younger males appeared at the dichotomy of 13 years (*p* = 0.001) and was maintained in subsequent dichotomies (*p* < 0.001 for all dichotomies of 14, 15, and 16 years). On the contrary, the QTcI difference between younger and older females was not significant for all dichotomies 10–15 years and became only significant at the dichotomy of 16 years (*p* = 0.014).

### QT/RR Dependency

By definition, both linear and log-linear regressions are bound to lead to larger QT/RR residuals compared to the curvilinear regressions (that is, the linear and log-linear QT/RR regression models cannot fit the data as closely as the curvilinear regressions). Nevertheless, in both sexes, the increases of QT/RR residuals from curvilinear to linear models (0.16 ± 0.30 ms and 0.12 ± 0.24 ms in females and males, respectively) were significantly smaller compared to the residual increases from curvilinear to log-linear models (0.89 ± 0.63 ms and 0.65 ± 0.30 ms in females and males, respectively; *p* < 001 for comparisons in both sexes). The increases of the residuals from the curvilinear models were also significantly smaller in males compared to females (*p* = 0.02 for linear models, *p* < 0.001 for log-linear models).

**FIGURE 7:**
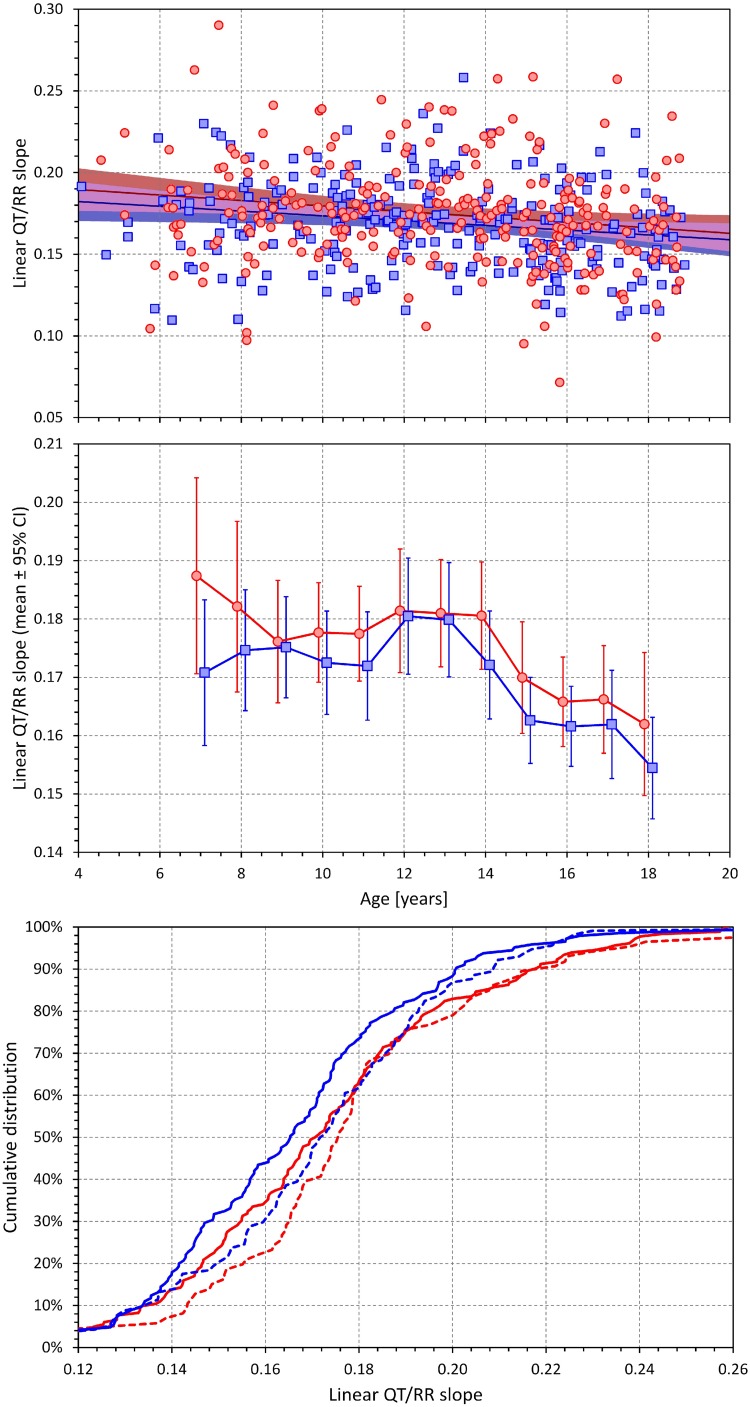
Development of the age dependency of linear QT/RR slopes in both sexes. The layout and meaning of the three panels are the same as the layout and meaning of the three panels of Figure 6.

Consequently, the linear models described the individual QT/RR relationships better compared to log-linear models and were used in the analysis of age dependency. The corresponding results are shown in Figure 7. In both sex groups, the QT/RR patterns became gradually shallower with advancing age (for both sexes *p* < 0.005; linear regression of QT/RR slopes vs. age). The middle panel of Figure 7 shows that this age effect was again non-linear with the age effect visible in both sexes from approximately 13 years onward. The bottom panel of Figure 7 shows sex-specific trends to shallower QT/RR slopes in subjects with secondary sex signs (compared to those without the signs) as well as trends to shallower QT/RR slopes in males compared to females (irrespective of the secondary sex signs) but none of these trends was statistically significant.

**FIGURE 8:**
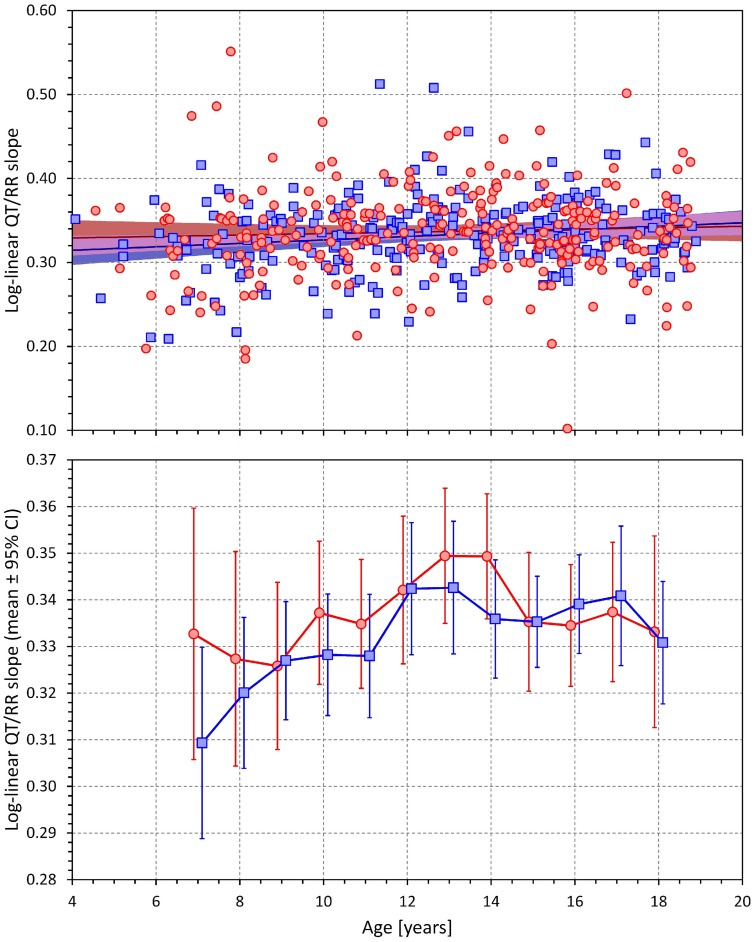
Age dependency of the slopes of the log-linear QT/RR regressions. The layout of the panels is the same as of the top two panels of Figure 6.

Corresponding analysis of the log-linear slopes is shown in Figure 8. This shows that the log-linear slopes led to erratic results, probably caused by the significantly lesser precision of the log-linear analysis.

## Discussion

**FIGURE 9:**
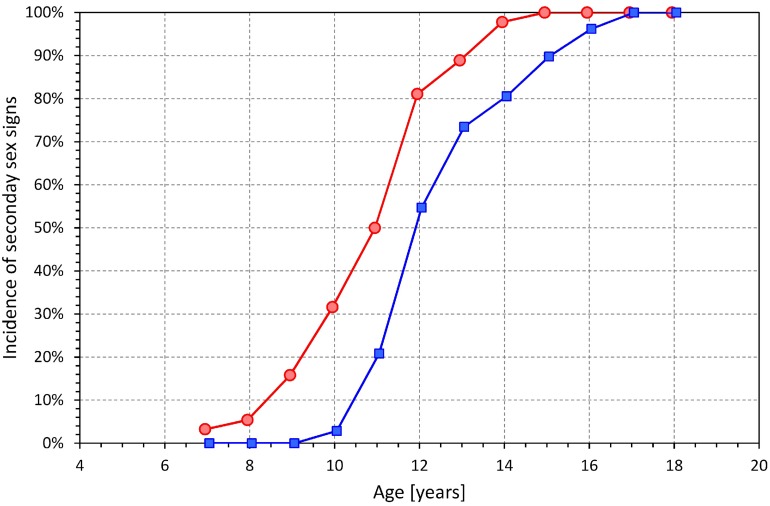
Incidence of secondary sex signs in age bands <8 years, 7–9 years, 8–10 years, etc. up to 16–18 years, and >17 years (each shown approximately at the middle age of the band). Compare with the development of QTcI changes shown in the middle panel of Figure 6.

The results of the study show that during puberty, the known sex difference in QTc interval does not appear at the same time as secondary sex signs. Whilst, as expected, we observed earlier onset of secondary sex signs in females compared to males (Mickey and Brooke, 2019), statistically significant QTc changes in females occurred some 3 years later compared to males (in addition to the statistical tests presented, compare also the middle panel of Figure 6 with Figure 9 that shows the development of incidence of secondary sex signs). Purely speculatively, this may suggest that simple hormonal shifts during puberty might be responsible for the QTc shortening in males but are unlikely the principal direct cause of QTc prolongation in females. If our observation is independently confirmed, other mechanisms need to be considered and researched, including the long-term pubertal conditioning, prolonged adaptation to perioding menstruation blood loss, or central and autonomic regulation changes. Indeed, the stability of menstrual cycle appears also after a considerable delay, perhaps similar to the delay that we observed with QTc prolongation, after the appearance of menarche (Lenton et al., 1984; Apter et al., 1987). Thus, while hormonal shifts might still contribute to the QTc prolongation in females, the mechanisms responsible for these repolarization changes are likely more intricate compared to the QTc shortening in males.

The study also shows that with simple postural maneuvering, wide heart rate spans known from clinical studies in adults (Malik et al., 2012b) may also be achieved in relatively young children. This has implications for studying the subject-specific QT/RR profiles that might be helpful when judging borderline cases of QTc interval abnormalities.

While the QT interval and heart rate data of this study might theoretically be also analyzed using previously proposed general correction formulae (e.g., Bazett, Fridericia, Hodges, or Framingham corrections), we have intentionally omitted such analyses since they would not only be inaccurate but potentially also highly misleading. It has recently been demonstrated (Hnatkova et al., 2019) that in the presence of heart rate changes exceeding 10 bpm, the use of fixed heart rate corrections might be substantially erroneous. The individually corrected QTc values that we obtained in this population were derived from averages over heart rate spans that well exceeded this “safety” limit (see Figure 5). In addition, the age-related changes in slopes of the QT/RR profiles further suggest that the experience with QTc heart rate correction formulae obtained in adult patients cannot be directly translated to accurate studies and/or clinical evaluations in children. In particular, the significantly worse log-linear regression fits of QT/RR profiles show that formulae based on this type of regression (e.g., Bazett or Fridericia corrections) are potentially highly misleading in individual clinical cases of school-aged children. Also, at fast rates as observed in younger children, the log-linear QT/RR relationship breaks down because the short uncorrected QT interval measurements (Hnatkova et al., 2017) making the use of formulae based on this relationship even more problematic.

The observation that taking QT/RR hysteresis (Malik et al., 2008b; Gravel et al., 2018) into account decreases the QT/RR residuals and thus increases accuracy of the QTcI assessment is consistent with previous observations in adults. It was previously shown that considerations of QT/RR hysteresis are not only essential during episodes of heart rate changes (Malik et al., 2016; Hnatkova et al., 2019) but also valuable during episodes without any physical activity since psychologically driven fluctuations of heart rate can hardly ever be eliminated (Malik et al., 2016, 2018). In the present study, short-term variability of heart rate, likely influenced by mental reactions, was also present during the individual episodes of the investigative protocol. The observations shown in Figure 1 were therefore not driven solely by the implications of the postural changes.

In agreement with previously published adult data (Malik et al., 2004, 2012b, 2013), we found only miniscule curvilinear QT/RR residuals (see the bottom panel of Figure 3). This means, among others, that in separate study individuals, the individually corrected QTc interval was practically constant during the postural provocations (detailed comparison not shown) and that the minimal variability along the curvilinear QT/RR regressions was only caused by the measurement jitter due to recordings noise. The QTcI values shown in Figure 6 and Supplementary Table 2 are thus applicable to different parts of the investigation experiments. Thus, similar to adult data (Malik et al., 2008c, 2013), we found the QT interval duration to be practically exclusively determined by the underlying heart rate (including the considerations of the QT/RR hysteresis) during these experiments in children and adolescents. Note also that the intra-individual QTcI stability was achieved by the sufficiently large intra-individual heart rate ranges over which the QT measurements were made and over which the QT/RR regressions were calculated.

We are not aware of other studies of individual-specific QT/RR profiles in children and adolescents with which we could compare the results of this study. Nevertheless, number of comparisons with adult data are possible. The averaged hysteresis constant close to 2 min is the same as previously reported based on adult investigations (Franz et al., 1988; Malik et al., 2008b). Clinical investigations in young to middle-aged adults found similar spans of minimal to maximal heart rates in response to similar postural maneuvering (Malik et al., 2012b). Like the adult studies, the investigated children and adolescents showed remarkable inter-subject variability of QT/RR profiles (Batchvarov et al., 2002; Malik et al., 2002). The comparison between the linear and log-linear QT/RR regressions is also known to have led to similar conclusions in adult data (Malik et al., 2012a).

Although we do not have detailed measurements of heart sizes available, it is reasonable to expect that as the body enlarges with advancing age (see Supplementary Figures 1, 2) so does the heart and ventricular mass. Nevertheless, this obviously cannot explain the QTc discrepancy between the sexes since with advancing age, the QTc interval changes differently in females and males. Also, while simple heart size might have some physiologic implications for heart rate, the influence on QTc interval, if it exists, is bound to be more complex and likely multifaceted.

While we observed a slightly higher heart rates in females compared to males (see the top panel of Figure 5), the differences were much smaller than the sex distinction known in adult resting heart rates (Linde et al., 2018). The reasons are possibly similar to those for the discrepancy between the secondary sex-sign maturity in females and their QTc prolongation. Prolonged autonomic conditioning in menstruating females might be needed for the autonomic regulatory equilibrium (Smetana and Malik, 2013) responsible for increased heart rates in adult pre-menopausal females.

The QTcI calculation was based on the projection of intra-subject curvilinear QT/RR regressions to predict the QT interval duration at a stable heart rate of 60 bpm. While this projection corresponded to the standard practice of heart rate correction, it required extrapolation of available data in younger children in whom the minimum heart rate was much higher (see the top panel in Figure 5). The low regression residuals (obtained when considering QT/RR hysteresis) suggest that this process did not lead to any substantial imprecision but in future studies in pediatric populations, corrections of QT interval to a different heart rate (e.g., 80 bpm) might prove somewhat more reliable.

### Limitations

Limitations of the investigation also need to be considered. While we collected demographic data and made sure that only normal subjects without clinically apparent abnormalities were included in the analysis, we were unable (for funding reasons) to subject the participants to further testing such as echocardiography, biochemistry, or detailed anthropometric measurements. For the same reasons, we were unable to collect continuous blood pressure data. Single instance blood pressure measurements were not collected since in children, the “white coat” effects are highly noticeable. This prevents us from considering renin-angiotensin regulation. For ethical reasons, we were also unable to collect any data on mental comprehension that might potentially be important for the development of autonomic conditioning. Nevertheless, as far as we can tell, it was unlikely that cognitions skills among the participants would have shown any sex-related or age-related bias outside the standard expectations of human development. For practicality reasons, it was also impossible to synchronize the investigations with a particular phase of menstrual cycle in menstruating females. Nevertheless, data on the last menstruation were also collected and when it was attempted to analyze the data considering the menstruation cycle, no meaningful influence was found (data not shown). Our observations of a very tight relationship between the QT interval duration and the underlying (hysteresis corrected) heart rate were made during awake state. We cannot comment on the QT/RR relationship in children during sleep which in known to influence QTc duration in adults (Stramba-Badiale et al., 2000; Lanfranchi et al., 2002). Finally, while the accuracy of RR interval histories of QT interval measurements was visually validated and manually corrected where necessary, the measurements of QT intervals relied on automatic computerized processing with exclusion of noise polluted ECG episodes. Nevertheless, the markedly low QT/RR regression residuals showed that the automatic QT interval measurement was fully reliable.

## Conclusion

Despite these limitations, the study shows that opposite QTc changes occur in both sexes during adolescent years. In the absence of detailed hormone level measurement, we can only speculate that while these QTc changes might be directly maintained by sex hormones in males, pure and direct sex hormone influence in females is less likely. The study also shows that tightly defined QT/RR patterns are achievable in children and adolescents by non-invasive postural testing. Since projection of such patterns may be used to compare the QT interval durations between different subjects, the described technique might be helpful in judging clinical cases of borderline QT interval abnormalities.

## Data Availability

All datasets generated for this study are included in the manuscript and/or the Supplementary Files.

## Author Contributions

MM, TN, and IA conceived the study. IA, KHn, TN, and MM designed the study. IA, KHe, MŠ, and TN contributed to the clinical conduct. TN and PK supervised the clinical conduct. KHn and MM analyzed the data. IA, KHn, and MM drafted the manuscript. All authors approved the final manuscript.

## Conflict of Interest Statement

The authors declare that the research was conducted in the absence of any commercial or financial relationships that could be construed as a potential conflict of interest.

## Footnotes

1 https://www.crediblemeds.org
