# Supplementary material for: Individually Rate Corrected QTc Intervals in Children and Adolescents
Source: Front Physiol. 2019 Aug 2;10:994. doi: 10.3389/fphys.2019.00994 (PMC6688657; doi:10.3389/fphys.2019.00994)
Supplement: Supplementary file 1 [file Table_1.DOCX]

Supplementary Figure 1


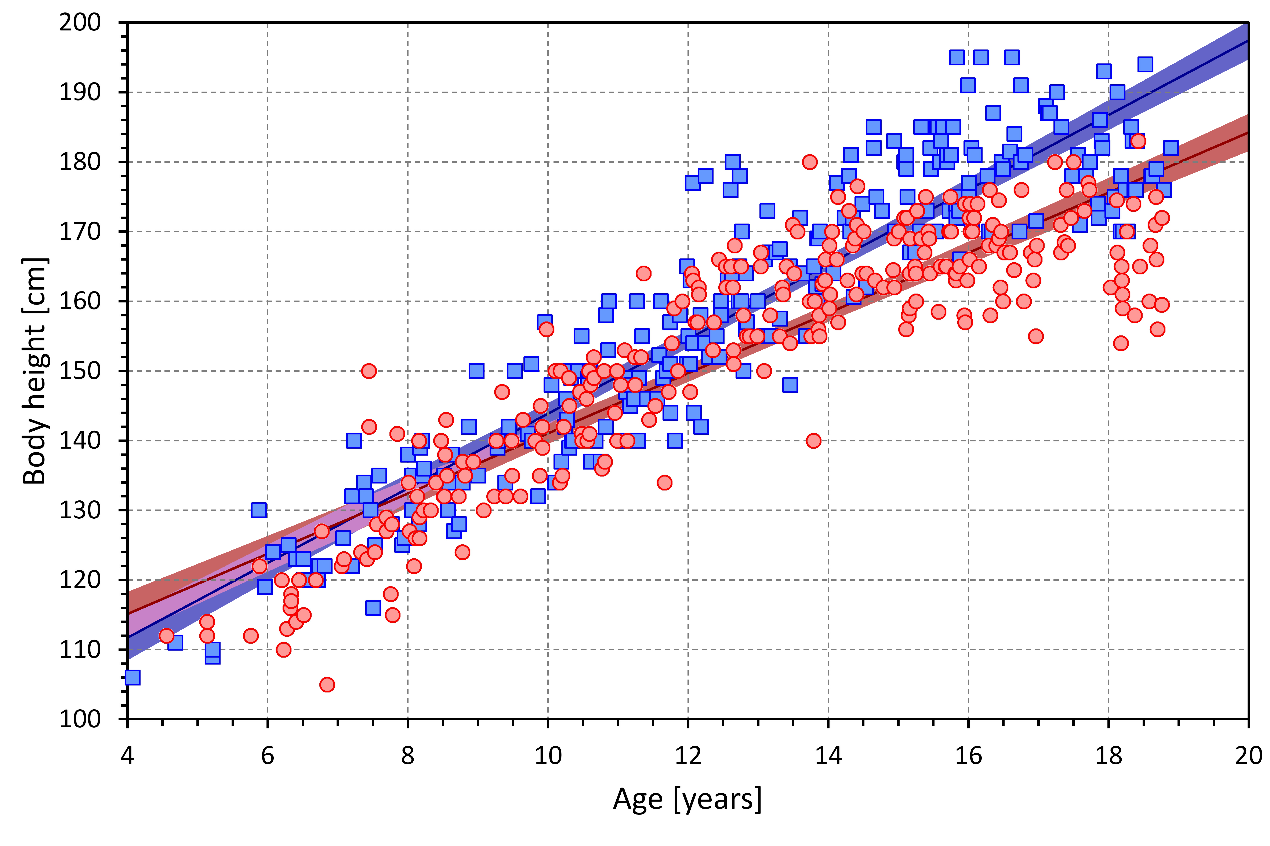

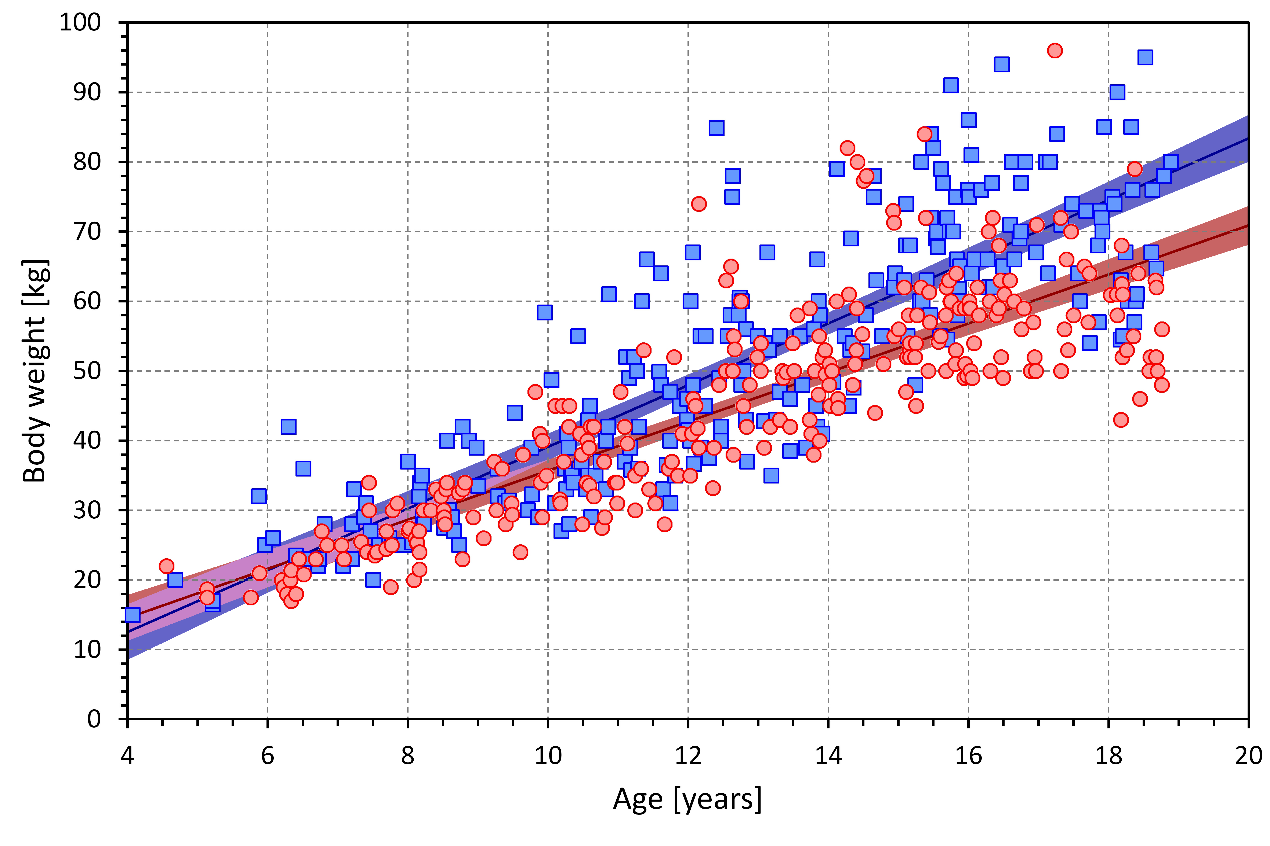


Age dependency of body height (top panel) and or body weight (bottom panel) in the investigated population. The red circles and blue squares correspond to the female and male subjects, respectively. The dark red and dark blue lines are linear regressions in females and males, respectively; the light red and light blue areas are the 95% confidence bands of the sex-specific linear regression lines. The violet areas are the overlaps between the confidence bands of the regression lines of both sexes.

Supplementary Figure 2


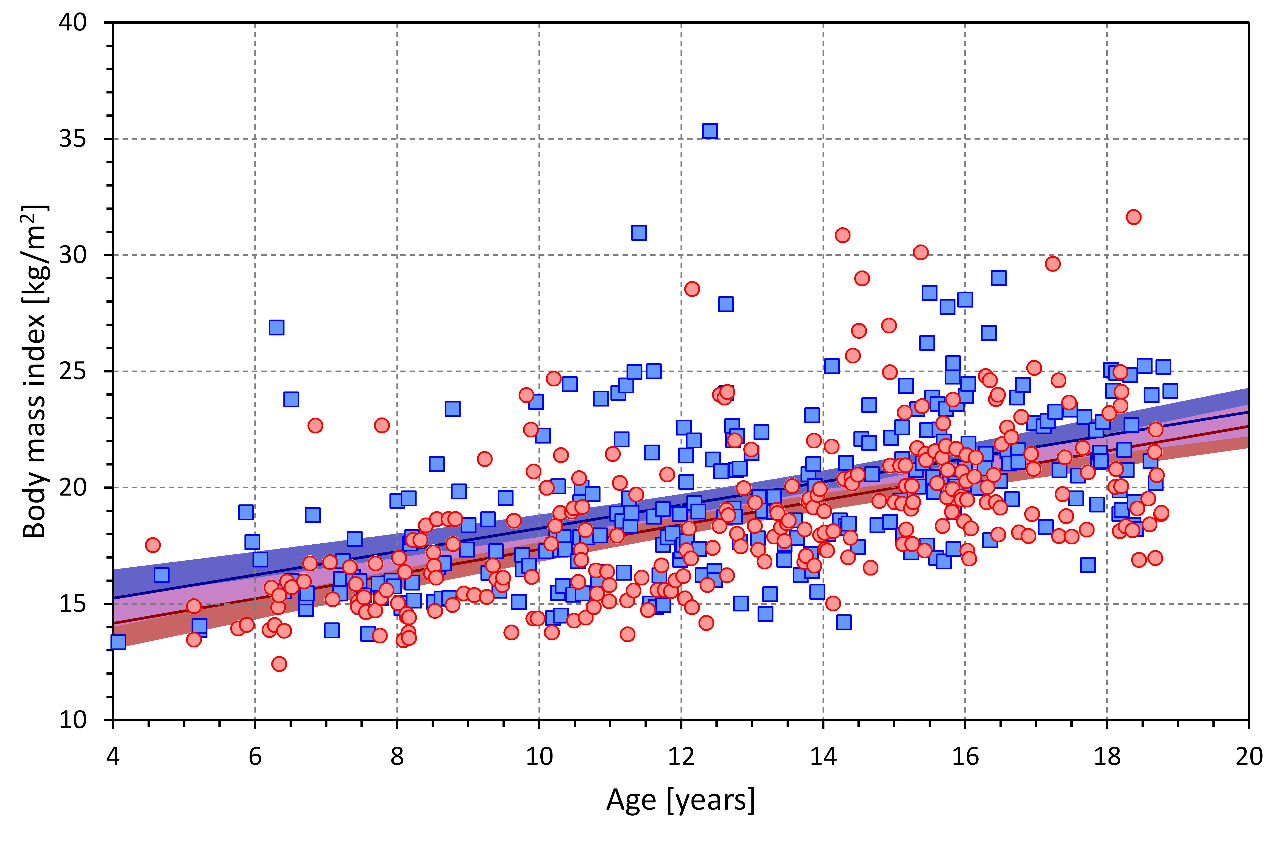


Age dependency of body mass index. The layout of the figure is the same as in the Supplementary Figure 1.

Supplementary Table 1

Heart rates in the investigated population

| Age [years] | **Females** | | | **Males** | | |
| --- | --- | --- | --- | --- | --- | --- |
|  | Minimum  heart rate | Maximum  heart rate | Heart rate  range | Minimum  heart rate | Maximum  heart rate | Heart rate  range |
| ≤7 | 80.4 ± 7.2 | 124.4 ± 11.7 | 44.1 ± 12.3 | 81.1 ± 5.8 | 123.1 ± 11.0 | 42.0 ± 8.7 |
| 7 to 8 | 82.3 ± 8.5 | 131.1 ± 15.9 | 48.8 ± 11.7 | 77.1 ± 7.3 | 122.9 ± 11.1 | 45.9 ± 9.6 |
| 8 to 9 | 74.5 ± 8.7 | 122.3 ± 11.7 | 47.8 ± 10.1 | 72.6 ± 5.8 | 123.1 ± 8.0 | 50.6 ± 8.6 |
| 9 to 10 | 71.1 ± 8.5 | 126.4 ± 12.4 | 55.4 ± 13.6 | 69.5 ± 8.1 | 122.8 ± 11.1 | 53.3 ± 12.6 |
| 10 to 11 | 69.9 ± 6.7 | 121.6 ± 13.9 | 51.7 ± 12.9 | 70.2 ± 8.0 | 121.7 ± 16.2 | 51.4 ± 13.0 |
| 11 to 12 | 71.4 ± 10.9 | 133.6 ± 15.8 | 62.2 ± 17.0 | 67.5 ± 9.1 | 124.2 ± 12.0 | 56.7 ± 14.0 |
| 12 to 13 | 68.0 ± 7.6 | 127.8 ± 12.7 | 59.8 ± 13.5 | 66.4 ± 8.9 | 127.6 ± 15.2 | 61.2 ± 14.3 |
| 13 to 14 | 64.1 ± 7.8 | 124.0 ± 15.9 | 59.9 ± 13.9 | 65.9 ± 9.1 | 119.7 ± 14.4 | 53.8 ± 12.5 |
| 14 to 15 | 65.8 ± 12.0 | 126.7 ± 14.9 | 60.9 ± 14.9 | 60.7 ± 8.0 | 126.6 ± 15.3 | 65.9 ± 13.0 |
| 15 to 16 | 62.2 ± 10.3 | 117.7 ± 16.2 | 55.5 ± 14.0 | 59.2 ± 8.6 | 114.6 ± 15.8 | 55.4 ± 12.3 |
| 16 to 17 | 60.2 ± 7.6 | 115.2 ± 12.0 | 55.0 ± 10.5 | 58.0 ± 7.9 | 110.8 ± 16.2 | 52.8 ± 14.3 |
| 17 to 18 | 59.7 ± 11.6 | 117.5 ± 13.6 | 57.8 ± 22.2 | 56.1 ± 7.7 | 118.6 ± 13.9 | 62.5 ± 10.2 |
| >18 | 57.2 ± 7.3 | 111.1 ± 13.7 | 54.0 ± 14.1 | 57.1 ± 7.9 | 115.0 ± 12.5 | 57.8 ± 10.7 |

For each age bin, the table shows the minimum and maximum heart rate and the within subject heart rate range. Data are in beats per minute and are shown as mean ± standard deviation.

Supplementary Table 2

QT/RR measurements in the investigated population

| Age [years] | **Females** | | | | **Males** | | | |
| --- | --- | --- | --- | --- | --- | --- | --- | --- |
|  | QTcI  Interval [ms] | QT/RR  Hysteresis [s] | QT/RR slope | QT/RR  residual [ms] | QTcI  Interval [ms] | QT/RR  Hysteresis [s] | QT/RR slope | QT/RR  residual [ms] |
| ≤7 | 401.2 ± 23.0 | 108.1 ± 25.6 | 0.178 ± 0.037 | 3.45 ± 0.82 | 389.8 ± 12.0 | 109.8 ± 16 | 0.164 ± 0.03 | 2.85 ± 0.52 |
| 7 to 8 | 413 ± 30.0 | 104.4 ± 16.9 | 0.199 ± 0.054 | 3.80 ± 0.65 | 411.4 ± 16.7 | 107.4 ± 15.7 | 0.178 ± 0.036 | 3.98 ± 1.51 |
| 8 to 9 | 408.5 ± 12.8 | 107.4 ± 24.9 | 0.172 ± 0.034 | 4.23 ± 1.51 | 405.2 ± 18.1 | 108.9 ± 14.6 | 0.172 ± 0.023 | 3.44 ± 0.80 |
| 9 to 10 | 412.0 ± 22.2 | 115.1 ± 23.4 | 0.183 ± 0.029 | 3.74 ± 0.66 | 410.5 ± 17.5 | 106.8 ± 15.8 | 0.180 ± 0.023 | 3.80 ± 1.20 |
| 10 to 11 | 409.2 ± 13.4 | 112.5 ± 20.5 | 0.174 ± 0.024 | 4.74 ± 2.75 | 405.9 ± 15.4 | 104.0 ± 16.7 | 0.169 ± 0.027 | 3.43 ± 0.61 |
| 11 to 12 | 411.4 ± 14.0 | 101.3 ± 23.3 | 0.182 ± 0.026 | 4.57 ± 3.25 | 410.9 ± 19.7 | 111.6 ± 13.5 | 0.175 ± 0.037 | 4.07 ± 1.40 |
| 12 to 13 | 408.9 ± 22.0 | 105.5 ± 18.4 | 0.181 ± 0.036 | 4.28 ± 1.97 | 414.6 ± 14.1 | 102.3 ± 20.9 | 0.185 ± 0.035 | 4.26 ± 0.97 |
| 13 to 14 | 415.2 ± 18.4 | 101.7 ± 23.4 | 0.181 ± 0.026 | 5.93 ± 3.35 | 411.4 ± 20.2 | 109.6 ± 19.6 | 0.172 ± 0.031 | 4.07 ± 1.29 |
| 14 to 15 | 413.5 ± 18.6 | 99.8 ± 23.1 | 0.180 ± 0.035 | 4.50 ± 2.25 | 406.0 ± 19.2 | 106.0 ± 17.7 | 0.172 ± 0.022 | 4.30 ± 1.76 |
| 15 to 16 | 408.9 ± 15.0 | 104.3 ± 20.9 | 0.163 ± 0.035 | 5.35 ± 3.44 | 399.0 ± 10.8 | 113.7 ± 23.0 | 0.158 ± 0.026 | 3.75 ± 1.02 |
| 16 to 17 | 412.1 ± 14.7 | 99.3 ± 26.7 | 0.169 ± 0.024 | 4.44 ± 1.97 | 404.5 ± 15.3 | 111.7 ± 15.9 | 0.168 ± 0.022 | 4.33 ± 1.25 |
| 17 to 18 | 415.9 ± 17.5 | 102.7 ± 22.6 | 0.160 ± 0.039 | 5.96 ± 5.76 | 395.4 ± 13.0 | 99.8 ± 12.2 | 0.155 ± 0.032 | 4.17 ± 1.28 |
| >18 | 417.6 ± 12.7 | 106.3 ± 18.0 | 0.163 ± 0.032 | 4.14 ± 0.90 | 390.7 ± 11.9 | 109.6 ± 18.1 | 0.154 ± 0.019 | 3.69 ± 0.53 |

For each age bin, the table shows the QTcI interval durations; the QT/RR hysteresis time-constant, i.e. the time needed for the 95% adaptation of the QT interval duration to be reached after a heart rate change; the curvilinear QT/RR slope; and the curvilinear QT/RR regression residual. Data are shown as mean ± standard deviation.
